# Transcriptomic and Physio-Biochemical Responses of the Fifth-Instar Larvae of *Chilo sacchariphagus* to High-Temperature Stress

**DOI:** 10.3390/insects17090907

**Published:** 2026-08-29

**Authors:** Ji-Li Wei, Feng-Ying Wang, Yong-Lin Ma, Xian-Kun Shang, Xue-Hong Pan, Ren-Zhao Liao, Liu-Feng Li, Qiao-Xian Wei

**Affiliations:** 1 Plant Protection Research Institute, Guangxi Academy of Agricultural Sciences, Nanning 530007, China; wjl2004-7919@163.com (J.-L.W.);; 2 Key Laboratory of Green Prevention and Control on Fruits and Vegetables in South China, Ministry of Agriculture and Rural Affairs, Nanning 530007, China; 3 Guangxi Key Laboratory of Biology for Crop Diseases and Insect Pests, Nanning 530007, China; 4 Sugarcane Research Institute, Guangxi Academy of Agricultural Sciences, Nanning 530007, China

**Keywords:** *Chilo sacchariphagus*, thermal tolerance in insects, transcriptomics, oxidative stress, antioxidant enzymes, heat shock protein, post-translational regulation

## Abstract

*Chilo sacchariphagus* is a destructive sugarcane borer worldwide, and frequent extreme high temperatures disrupt its field populations. Clarifying larval thermal response mechanisms provides theoretical support for pest risk prediction under climate warming. Previous work has illustrated moderate heat induces canonical HSP activation, but the molecular responses of this pest to 41 °C extreme heat remain unclear. As such, we conducted non-reference transcriptome sequencing and 11 physio-biochemical assays on fifth-instar larvae exposed to 41 °C for 12 h (26 °C as control) to characterize its thermal regulatory network. De novo assembly yielded a comprehensive transcriptome resource, and analysis of differentially expressed genes revealed enrichment in energy metabolism, ER protein processing, MAPK signaling and autophagy pathways. Core HSP70/40 transcripts were significantly down-regulated while HSP80 showed stable transcription, suggesting that 41 °C may exceed the heat-shock protective threshold and potentially trigger heat damage. qRT-PCR validation of seven core stress genes confirmed the RNA-seq trends. Catalase (CAT) activity increased significantly; however, none of the annotated catalase genes showed transcriptional changes, whereas elevated CarE and AchE activities also lacked corresponding transcriptional shifts, implying post-translational or alternative regulatory mechanisms. Our data showed coordinated transcriptional and physiological changes under extreme heat. These findings provide a basis for further investigation into how *C. sacchariphagus* may respond to extreme heat under climate warming scenarios.

## Simple Summary

*Chilo sacchariphagus* is a globally important sugarcane borer. Characterizing its responses to extreme heat aids projections of pest dynamics amid climate warming. Integrated transcriptomic and physio-biochemical assays at 41 °C showed selective down-regulation of HSP70/40 while HSP80 transcription remained stable. Despite a significant increase in catalase (CAT) enzyme activity, none of the annotated catalase genes showed transcriptional changes, suggesting that this increase may involve post-translational regulation or other mechanisms. These findings indicate that extreme heat appears to exceed the insect’s normal protective capacity, triggering alternative defense responses that may influence its adaptation under future climate warming scenarios.

## 1. Introduction

Sugarcane (*Saccharum* spp.) is a major sugar crop and strategic commodity in China. Guangxi, as the largest sugar-producing base in the country, produced 6.465 million tons of sugar in the 2024/2025 crushing season, ranking first in the nation for 34 consecutive seasons and accounting for about 60% of China’s total output [1]. As an important global economic crop, sugarcane is not only the primary source of sugar but also plays key roles in energy, food, and other fields [2,3,4]. Sugarcane is a pillar industry for the economic development of Guangxi and an important guarantee for China‘s sugar security. *Chilo sacchariphagus* (Bojer, 1856) (Lepidoptera: Crambidae) is one of the most destructive boring pests of sugarcane in Guangxi [5,6]. According to monitoring by the Guangxi Plant Protection Station in 2025, the first generation of stem borers caused serious damage in some sugarcane areas of southern Guangxi, with dead-heart rates ranging from 12.5% to 18.6% and reaching up to 41.3% in individual fields. In the Beihai sugarcane region of Guangxi, sex pheromone trapping during the seedling stage showed that *C. sacchariphagus* accounted for over 50% of the total male moth catches, significantly outnumbering other borer species and indicating its clear population dominance during the early growth phase of sugarcane [7]. The larvae bore into the stalks at an early stage and feed internally, making this pest difficult to control and resulting in substantial economic losses to sugarcane production. This pest has therefore become a major constraint to the sustainable and high-efficiency production of sugarcane in the region.

Temperature is a key ecological factor affecting insect development, growth, and reproduction. Elevated temperatures not only influence the development of individual organisms but may also disrupt interspecific relationships such as competition, predation, and parasitism, thereby having profound consequences for biodiversity and ecosystem functioning [8,9]. As ectothermic organisms, insects are highly sensitive to temperature changes in their growth, development, and reproduction [10,11]. The sensitivity of the insect reproductive system to temperature variation has been confirmed by multiple studies [12,13]. Abnormal increases in temperature may alter the reproductive behavior of *C. sacchariphagus*, thereby affecting its population dynamics and the extent of crop damage. Previous studies have demonstrated that temperature significantly influences the developmental duration, survival rate, and fecundity of *C. sacchariphagus* at different life stages. Within the range of 20–32 °C, the duration of each developmental stage decreases with increasing temperature, and the optimum temperature range for development and reproduction is 26–29 °C [14]. However, the mechanisms by which extreme high temperatures affect the population dynamics of this pest remain unclear. Notably, spring in 2025 was characterized by drought conditions in Guangxi, and studies have shown that drought stress significantly aggravates the population growth and damage caused by sugarcane borers [15]. At the same time, extreme high-temperature events have become more frequent with global warming. High-temperature weather in Guangxi is characterized by wide coverage, long duration, and strong extremity [16]. In 2023, many areas recorded extreme high temperatures above 41 °C, and the temperature remained relatively high in 2024. The combined effects of drought and heat on the population dynamics of *C. sacchariphagus* remain largely unknown.

Insects have evolved multiple molecular strategies to cope with high-temperature stress. Heat shock proteins (HSPs) act as molecular chaperones that play a central role in the cellular stress response by assisting protein folding, preventing aggregation, and maintaining protein homeostasis [17]. However, the protective role of HSPs has an upper temperature limit; when temperatures exceed the effective induction range, the HSP system itself becomes suppressed and loses its protective function—a stage considered to have entered the “heat-damage” phase [18,19]. For example, in *Bemisia tabaci* (Gennadius, 1889), hsp70 expression peaked at 41 °C and then decreased rapidly at higher temperatures [20], suggesting that 41 °C may represent a critical threshold for heat-induced injury in some insects. Similar complex patterns of HSP regulation have been observed in other species under various thermal regimes [21,22,23]. Beyond HSPs, the relationship between transcriptional and functional responses of protective enzymes under heat stress is often complex, as enzyme activity is not always predictable from transcript levels alone [24,25]. Transcriptome sequencing has emerged as a powerful approach for identifying candidate genes and pathways involved in stress responses, particularly when integrated with physiological measurements [26,27]. Despite these advances, the molecular response of *C. sacchariphagus* to extreme heat (41 °C) remains poorly understood.

The relationship between transcriptional and functional responses of protective enzymes under heat stress is often complex. For instance, in the two-spotted spider mite *Tetranychus urticae* (Koch, 1836), two catalase genes exhibited opposite expression patterns under heat stress—*TuCAT1* was up-regulated while *TuCAT2* was down-regulated, suggesting that catalase family members are differentially regulated at the transcriptional level [24]. Moreover, discrepancies between transcript abundance and protein function have been documented in other insects; in *Zeugodacus cucurbitae* (Coquillett, 1899), extreme heat significantly down-regulated a target gene at the mRNA level without altering its protein abundance [25]. Detoxification enzymes also show complex responses: carboxylesterase (CarE) activity in *Lymantria dispar* (Linnaeus, 1758) responded to thermal stress in a tissue-specific manner [28], while heat shock has been reported to induce acetylcholinesterase (AchE) expression in *Drosophila melanogaster* (Meigen, 1830) [29]. These findings indicate that enzyme activity under heat stress is not always predictable from transcript levels alone, and that post-transcriptional or post-translational mechanisms may play important roles in modulating physiological responses. Despite these complexities, transcriptome sequencing remains a powerful first-line approach for identifying candidate genes and pathways involved in stress responses, particularly when integrated with physiological measurements.

Despite these advances in other insect species, the molecular response of *C. sacchariphagus* to extreme heat (41 °C) remains poorly understood. Fifth-instar larvae of *C. sacchariphagus* may be significantly affected by heat treatment at 41 °C. To elucidate the molecular response mechanisms in this study, we performed transcriptome sequencing of fifth-instar larvae treated at 41 °C for 12 h (with 26 °C as control) and measured 11 physio-biochemical indices under different temperature regimes. Through the screening of differentially expressed genes, functional enrichment analyses, and identification of core genes, we systematically characterized the molecular regulatory network of fifth-instar *C. sacchariphagus* larvae in response to high-temperature stress. The results provide a mechanistic basis for understanding the impact of high-temperature stress on the population dynamics of this important sugarcane pest.

## 2. Materials and Methods

### 2.1. Insects

The experimental colony of *C. sacchariphagus* was reared on an artificial diet [30] for over 20 consecutive generations under controlled conditions: 26 ± 1 °C, 70 ± 5% relative humidity, and a photoperiod of 14 h light:10 h dark. Fifth-instar larvae of uniform size were used for all experiments.

### 2.2. High-Temperature Treatments

Fifth-instar larvae were exposed to 26 °C (control), 35 °C, 38 °C, or 41 °C for 12 h in an artificial climate chamber (RXZ-380C, Ningbo Jiangnan Instrument Factory, Ningbo, China) with temperature accuracy of ±0.5 °C under dark conditions and with food provided during the exposure. The 41 °C treatment was selected based on historical extreme temperature records in Guangxi sugarcane-growing areas [16], and the 12 h duration simulates a typical diurnal heatwave episode. Relative humidity (70 ± 5%) and photoperiod (14 h light:10 h dark) were maintained at constant. Each treatment consisted of three biological replicates (eight larvae per replicate). Immediately after treatment, all larvae (no mortality was observed in any temperature group during the 12 h exposure) were flash-frozen in liquid nitrogen and stored at −80 °C for subsequent analyses.

### 2.3. RNA Extraction and Quality Control

Total RNA was extracted using TRIzol reagent (Invitrogen, Carlsbad, CA, USA), yielding >2 µg per sample. RNA integrity was assessed by 0.8% agarose gel electrophoresis and spectrophotometry, and only RNA with an A_260_/_280_ ratio between 1.8 and 2.2 was used for library construction. cDNA libraries were prepared using an Illumina TruSeq kit (Illumina, San Diego, CA, USA) according to the manufacturer’s instructions, with oligo-dT primers for mRNA reverse transcription (APExBIO, Cat. No. K1159). Libraries were size-selected (350–550 bp), quality-assessed on an Agilent 2100 Bioanalyzer (Agilent, Santa Clara, CA, USA), and sequenced on an Illumina NovaSeq 6000 platform (Illumina, San Diego, CA, USA) (paired-end 150 bp) to generate raw reads (APExBIO Co., Ltd., Shanghai, China).

### 2.4. Data Processing, Assembly and Functional Annotation

Raw reads from the six samples were filtered using TrimGalore (version 0.6.4) to remove low-quality reads and adapter sequences. De novo transcriptome assembly was performed using Trinity (version 2.4.0) to generate transcripts and unigenes [31,32]. After redundancy removal, the resulting unigenes were further clustered using Corset (version 1.09) based on shared reads and expression levels across samples to generate gene-level clusters [33]. The assembled transcriptome served as the reference for read alignment using Bowtie (version 1.2.3), and unigene abundance was quantified with RSEM (version 1.3.3) [34,35]. Transcript counts were normalized to transcripts per million (TPM) [36]. Functional annotation was performed using the Trinotate suite (version 3.2.2) against the Nr, Pfam, and Swiss-Prot databases with an E-value cut-off of 1.0 × 10^−5^ [37]. GO and KEGG annotations were obtained via eggNOG-mapper (version 2.1.12) and GhostKOALA (version 3.0.3), respectively.

### 2.5. Differential Expression Analysis

Differential expression analysis was performed using the DESeq2 R package (version 1.28.1) on raw read counts [38]. The Benjamini–Hochberg method was used to adjust *p*-values. Genes with an adjusted *p*-value < 0.05 and |log_2_(fold change)| > 1 were considered differentially expressed. GO and KEGG enrichment analyses were performed using the clusterProfiler R package (version 3.16.1) [39].

### 2.6. Physio-Biochemical Measurements

Frozen fifth-instar larvae were weighed (0.1–0.2 g), homogenized on ice in 1 mL of extraction buffer, and centrifuged at 12,000 rpm for 10 min at 4 °C. The supernatant was collected for the following assays. All measurements were performed according to the manufacturer’s protocols (Nanjing Jiancheng Bioengineering Institute, Nanjing, China).

Superoxide dismutase (SOD) activity was measured using the WST-8 method at 450 nm; catalase (CAT) activity was determined by monitoring H_2_O_2_ decomposition at 240 nm; peroxidase (POD) activity was measured using guaiacol at 470 nm. CAT activity was expressed as μmol min^−1^ g^−1^ fresh weight, while SOD and POD activities were expressed as U g^−1^ fresh weight.

Glutathione S-transferase (GST) activity was assayed using the CDNB method at 340 nm; carboxylesterase (CarE) activity was determined using α-naphthyl acetate at 450 nm; acetylcholinesterase (AchE) activity was measured using the DTNB method at 412 nm. GST and AchE activities were expressed as nmol min^−1^ g^−1^ fresh weight, and CarE activity as U g^−1^ fresh weight.

Total protein content was determined by the Coomassie Brilliant Blue G-250 method at 620 nm; triglyceride (TG) content was assayed by the GPO-PAP enzymatic method at 505 nm; trehalose and glycogen were measured by the anthrone colorimetric method at 620 nm; sorbitol was determined by the copper ion complexation method at 655 nm. All contents were expressed as mg g^−1^ fresh weight.

All physio-biochemical data were analyzed using one-way ANOVA followed by Duncan‘s multiple range test (SPSS 26.0). A *p*-value < 0.05 was considered statistically significant.

### 2.7. qRT-PCR Validation

*β-Actin* was used as the internal reference gene [40]. Specific primers for seven core genes (*ACS*, *HSP7C*, *DNJH*, *SODC2*, *CYP1*, *CYP63*, *CATA2*) were designed and synthesized by Yikesong Biotechnology Co., Ltd. (Nanning, China) using Primer Premier 5.0 software. Primer sequences are listed in Table 1.

Total RNA was extracted from approximately 50 mg of frozen larvae using the TransZol Up Plus RNA Kit (TransGen Biotech, Beijing, China). RNA concentration and purity were measured spectrophotometrically. Reverse transcription was performed using the TransScript Uni All-in-One First-Strand cDNA Synthesis SuperMix (TransGen Biotech, Beijing, China) following the manufacturer‘s instructions, using 1 µg of total RNA. The resulting cDNA was diluted and stored at −20 °C.

qRT-PCR was performed using PerfectStart Green qPCR Super Mix (TransGen Biotech, Beijing, China) on a Bio-Rad CFX96 real-time PCR system (Bio-Rad, Hercules, CA, USA). β-Actin served as the internal control. The 20 µL reaction mix contained 10 µL of 2× qPCR Super Mix, 8 µL of diluted cDNA (~40 ng), and 0.4 µL each of forward and reverse primer (10 µM). The thermal cycling protocol was: 95 °C for 120 s; 40 cycles of 95 °C for 5 s and 60 °C for 30 s; followed by a melt curve analysis. All samples were run in three technical replicates. Relative expression levels were calculated using the 2^−ΔΔCT^ method with β-Actin as the reference. Data are presented as mean ± standard error of the mean (SEM). One-way ANOVA followed by Duncan‘s multiple range test was performed using SPSS 26.0, with *p* < 0.05 considered statistically significant.

## 3. Results

### 3.1. Quality Assessment of Transcriptome Sequencing Data

Transcriptome analysis was performed on larvae exposed to 26 °C (control) and 41 °C (heat treatment). Six samples were sequenced, generating a total of 350 million raw reads (52.6 Gb raw data) (Table 2). After filtering, 345 million clean reads (51.6 Gb clean data) were obtained, with an effective data rate above 98.39% for all samples. Quality assessment showed that the sequencing error rate was 0% for all samples; Q20 values ranged from 99.34% to 99.50%, Q30 values from 96.24% to 97.35%, and GC content was stable between 44.7% and 45.4%. These results indicate that the sequencing data are of high quality and suitable for subsequent analyses.

### 3.2. Transcriptome Assembly and Functional Annotation

De novo assembly with Trinity yielded 132,531 transcripts with a total length of 162,008,603 bp, an average length of 1222.42 bp, a median length of 656 bp, and an N50 of 2069 bp. After redundancy removal, 90,394 unigenes were obtained, with a total length of 95,359,879 bp, average length of 1054.94 bp, median length of 546 bp, and N50 of 1812 bp (Table 3).

BUSCO assessment against the endopterygota_odb10 database (version 5.3.2) revealed that 98.0% of the complete BUSCOs were recovered (C: 98.0% [S: 1.4%, D: 96.6%], F: 1.1%, M: 0.9%), indicating a high level of completeness of the transcriptome assembly.

After Corset clustering of the 90,394 assembled unigenes (Table 3) into gene-level clusters based on shared reads and expression profiles, a total of 89,352 unigenes were obtained and used for functional annotation (Table 4). Functional annotation against public databases revealed that 21,790 unigenes (24.4%) were annotated in NR, 15,545 (17.4%) in Pfam, 10,943 (12.3%) in GO, 5440 (6.1%) by BLASTX against Swiss-Prot, 4833 (5.4%) by BLASTP against Swiss-Prot, 3251 (3.6%) in eggNOG, and 1548 (1.7%) in KEGG.

**Figure 1:**
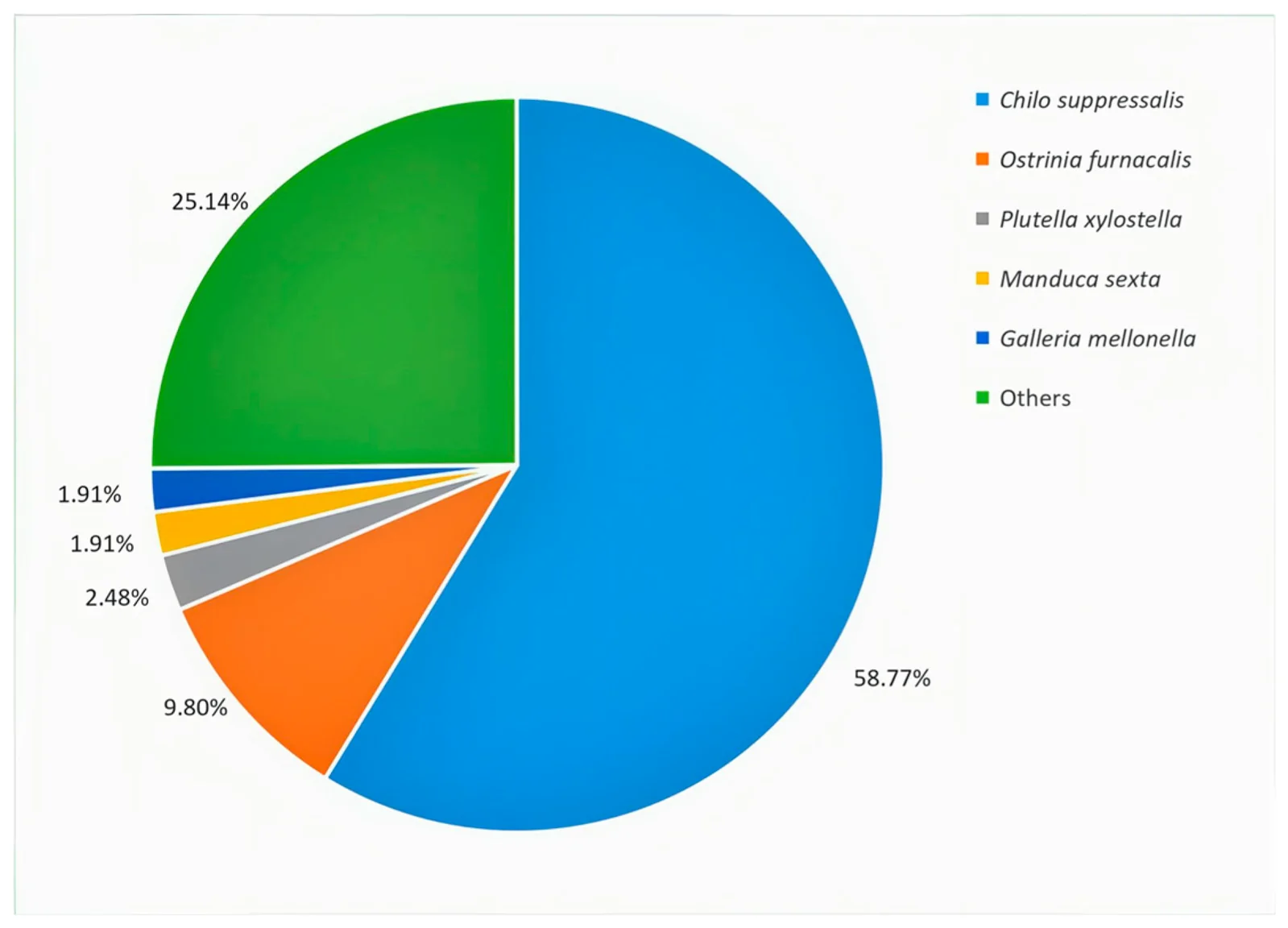
Species distribution of NR database annotations.

When unigene sequences were aligned against the NR database, the species distribution showed that most annotated genes belonged to lepidopteran insects (Figure 1). The highest similarity was with *Chilo suppressalis* (58.77%, same family Crambidae), followed by *Ostrinia furnacalis* (9.8%) and *Plutella xylostella* (2.48%). Other matches included *Manduca sexta* (1.91%) and *Galleria mellonella* (1.91%). This confirms the reliability of the sample origin and the high confidence of the functional annotation.

### 3.3. Overall Analysis of Differentially Expressed Genes

**Figure 2:**
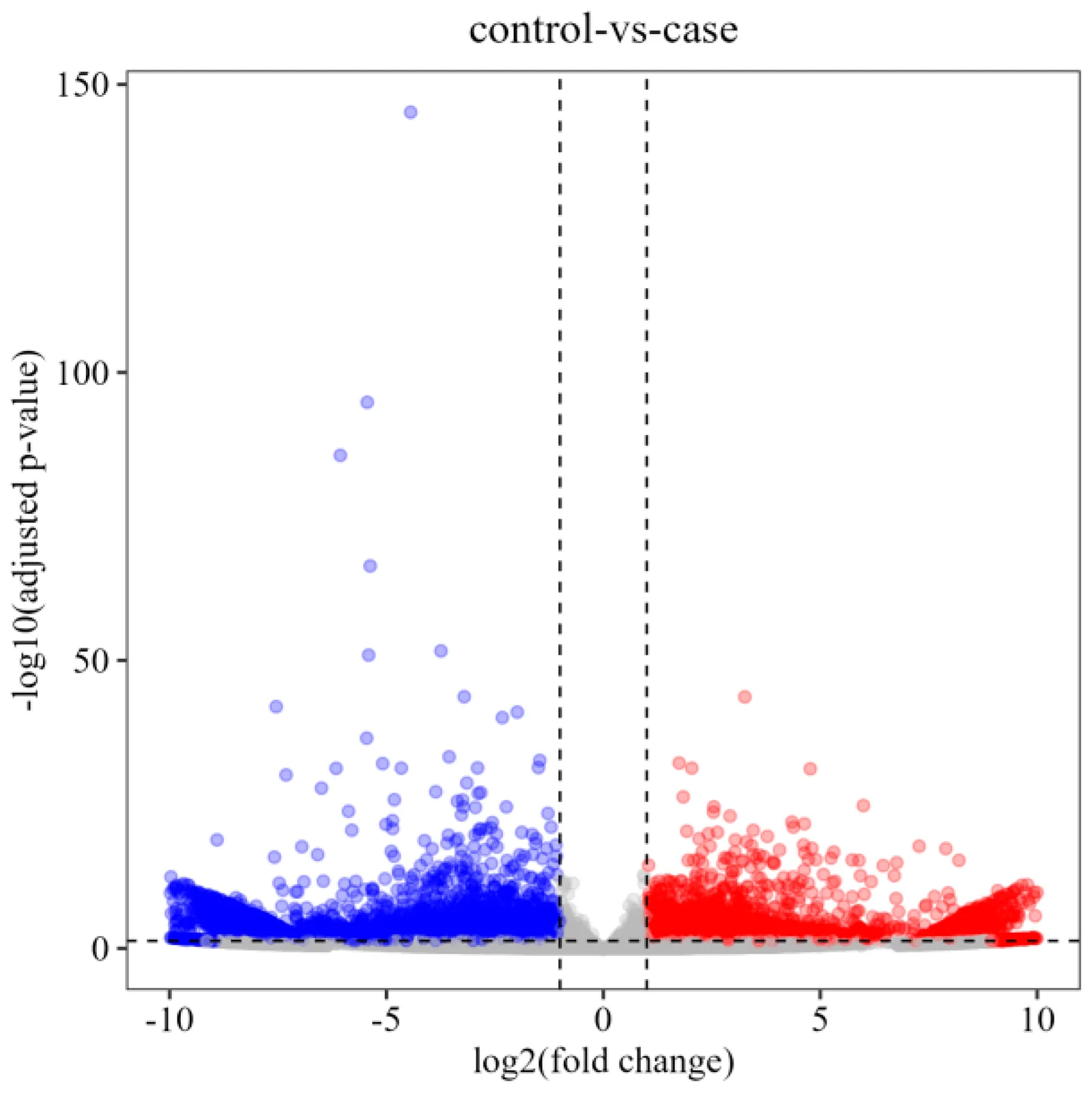
Volcano plot of differentially expressed genes. The x-axis represents log_2_(fold change) (positive = up-regulation, negative = down-regulation); the y-axis represents −log_10_(adjusted *p*-value). Red dots: significantly up-regulated genes; blue dots: significantly down-regulated genes; gray dots: non-significant genes. The horizontal dashed line indicates the significance threshold (adjusted *p*-value = 0.05), and the vertical dashed lines indicate the fold change threshold (|log_2_FC| = 1). Note: hyphens (-) in the figure indicate negative values.

**Figure 3:**
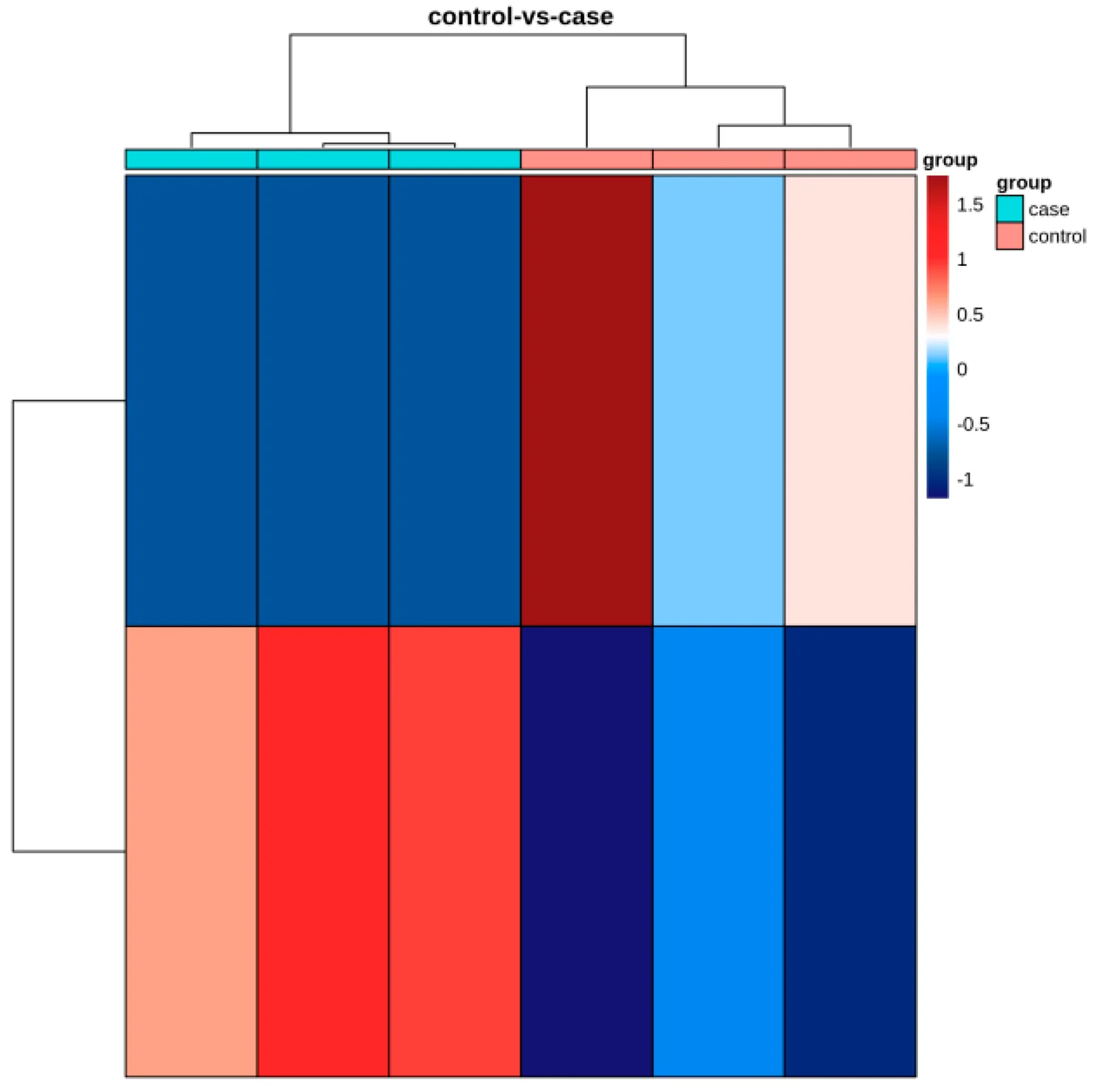
Heatmap of differentially expressed genes. Each row represents a gene, each column a sample. Color scale from blue to red indicates low to high expression levels. Cases 1–3: 41 °C treatment; controls 1–3: 26 °C control. Note: hyphens (-) in the figure indicate negative values.

Using a threshold of |log_2_FC| ≥ 1 and padj < 0.05, a total of 8563 DEGs were identified between the high-temperature (41 °C) and control (26 °C) groups, with 3352 up-regulated and 5211 down-regulated genes in heat-stressed larvae relative to controls (Table 5). The volcano plot shows the overall distribution of DEGs, where red and blue dots represent up- and down-regulated genes, respectively. Notably, down-regulated genes substantially outnumbered up-regulated genes under heat stress (Figure 2). Hierarchical clustering clearly separated the heat-treated samples from the controls, indicating that 41 °C stress induced extensive transcriptomic reprogramming in fifth-instar larvae of *C. sacchariphagus* (Figure 3).

### 3.4. GO Enrichment Analysis

**Figure 4:**
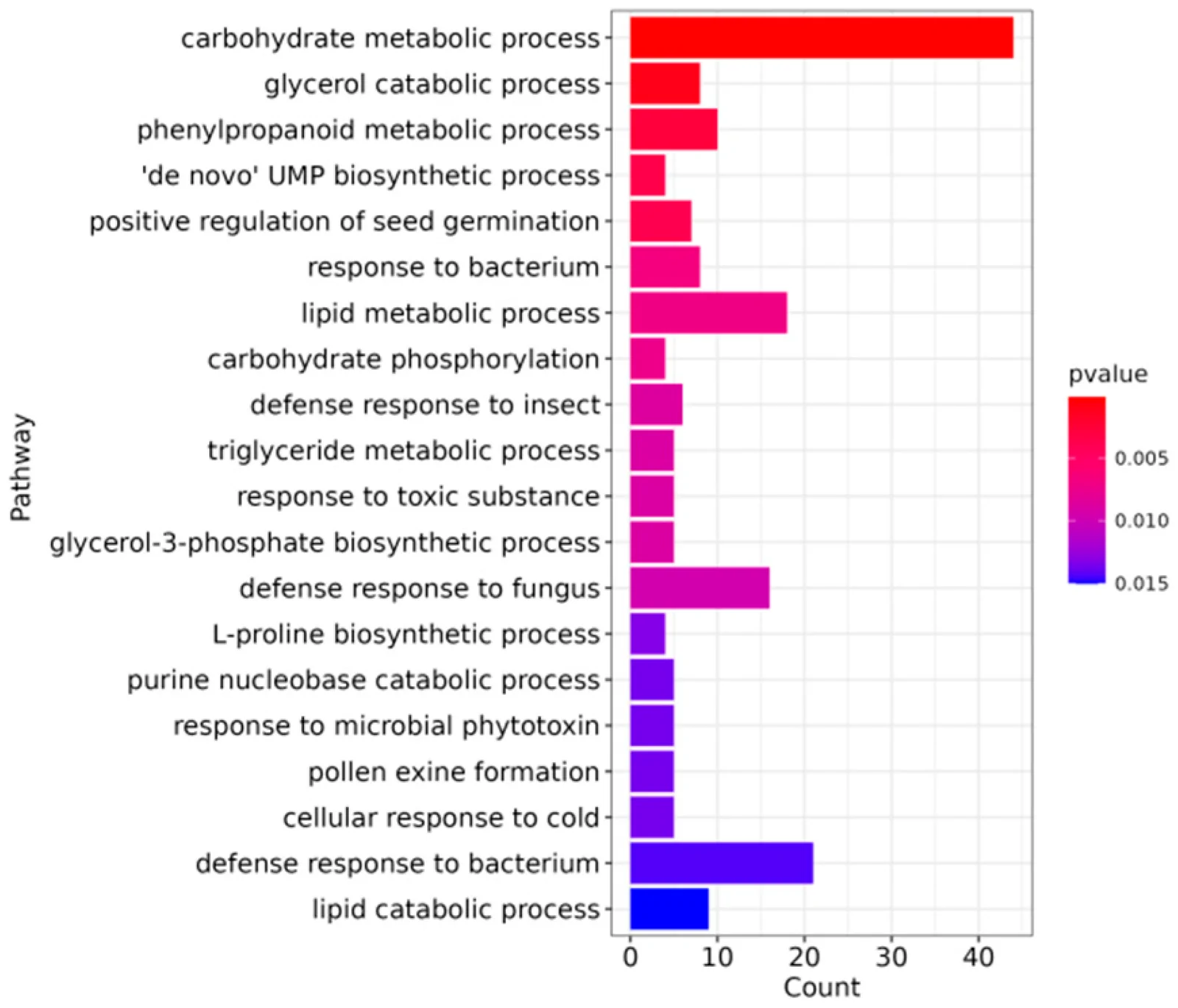
Top 20 GO biological process enrichment. The y-axis shows GO terms, the x-axis GeneRatio. Bar height represents gene count; color represents *p*-value (darker red = more significant).

GO enrichment analysis showed that DEGs were significantly enriched in multiple biological process terms (Figure 4). The enriched terms included energy-metabolism-related processes such as carbohydrate metabolic process, glycerol catabolic process, and lipid metabolic process. Stress-response terms including response to toxic substance, L-proline biosynthetic process, and defense response to bacterium were also significantly enriched. It should be noted that because functional annotation was based on BLASTX against the NR database, some plant-specific GO terms (e.g., ‘positive regulation of seed germination,’ ‘phenylpropanoid metabolic process,’ and ‘pollen exine formation’) appeared as annotation artifacts and were excluded from biological interpretation. The top 20 enriched GO terms are presented in Figure 4.

### 3.5. KEGG Pathway Enrichment Analysis

**Figure 5:**
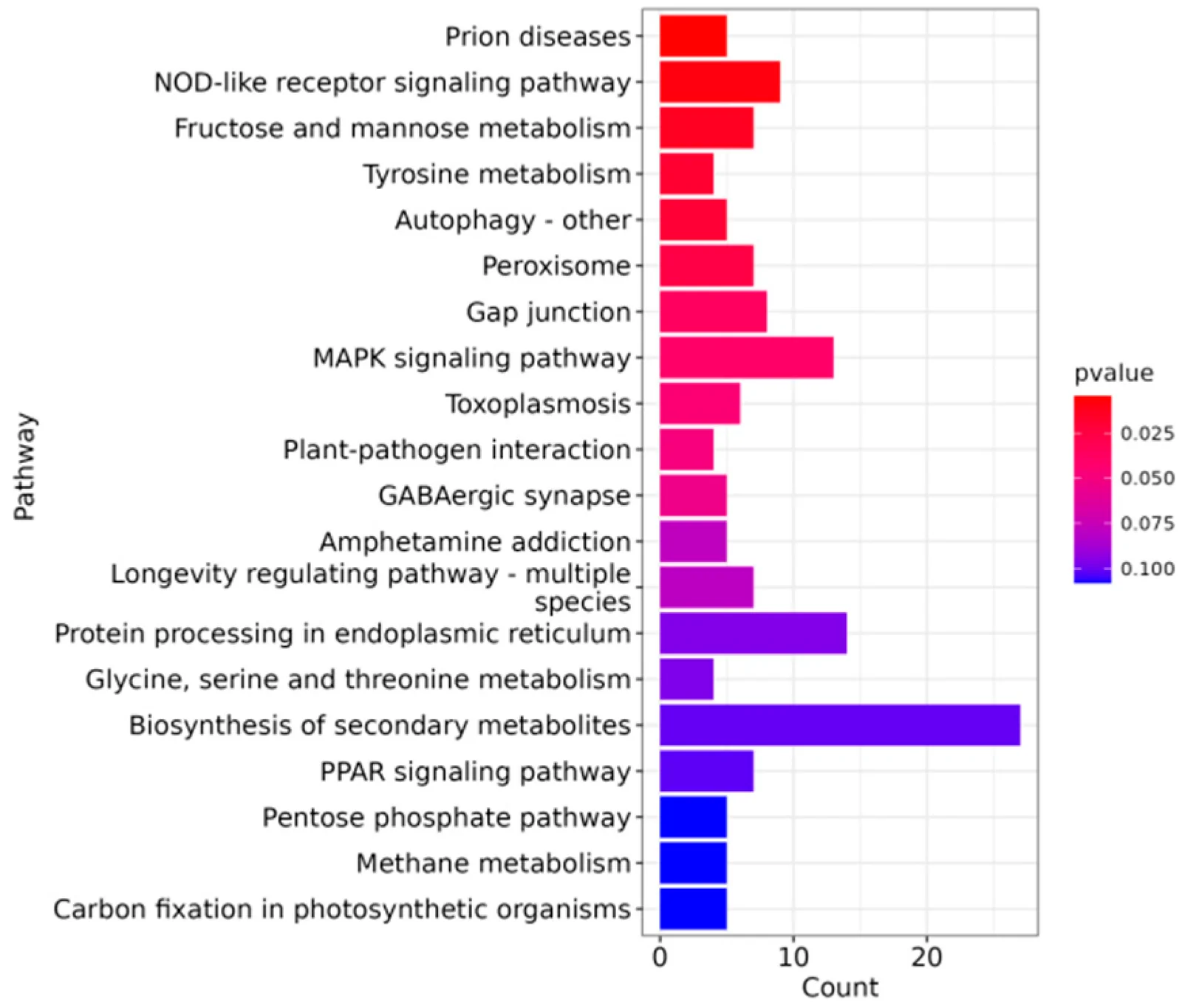
Top 20 KEGG pathway enrichment. The y-axis shows pathway names, the x-axis GeneRatio. Bar height represents gene count; color represents *p*-value (darker red = more significant).

KEGG enrichment analysis showed that DEGs were significantly enriched in multiple pathways (Figure 5). The enriched pathways included protein processing in endoplasmic reticulum, MAPK signaling pathway, peroxisome, autophagy, and several energy-metabolism pathways (e.g., fructose and mannose metabolism, tyrosine metabolism, glycine, serine and threonine metabolism, and PPAR signaling pathway). It should be noted that because functional annotation was based on BLASTX against the NR database, some plant-related KEGG terms (e.g., ‘plant–pathogen interaction’ and ‘carbon fixation in photosynthetic organisms’) appeared as annotation artifacts and were excluded from biological interpretation.

### 3.6. Effects of High-Temperature Stress on Physio-Biochemical Indices

**Figure 6:**
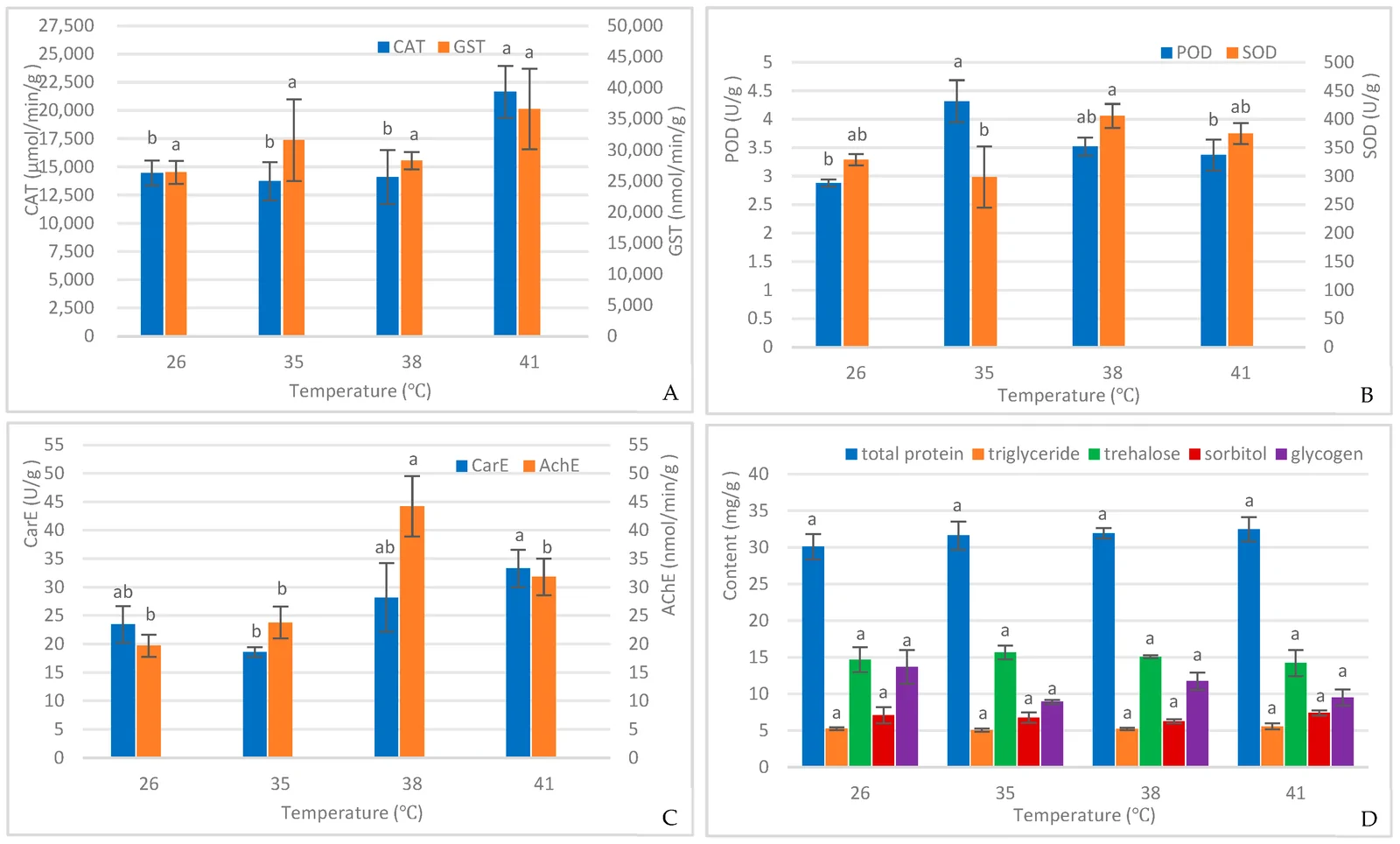
Changes in physiological and biochemical indices in fifth-instar larvae of *C. sacchariphagus* under different temperature stresses. (**A**) CAT (catalase) and GST (Glutathione S-transferase); (**B**) POD (peroxidase) and SOD (Superoxide dismutase); (**C**) CarE (carboxylesterase) and AchE (acetylcholinesterase); (**D**) total protein, triglyceride, trehalose, sorbitol and glycogen. Data are presented as mean ± standard error. Statistical significance was determined by one-way ANOVA followed by Duncan’s multiple range test (*p* < 0.05). Different lowercase letters indicate significant differences among temperature groups for the same index.

Physio-biochemical assays were conducted across all four temperature regimes (26, 35, 38, and 41 °C). Under high-temperature stress, CAT activity significantly increased only at 41 °C; GST activity showed no significant changes across all temperature treatments (Figure 6A); POD activity peaked at 35 °C, while SOD activity peaked at 38 °C; both showed lower activities at 41 °C (Figure 6B). CarE activity showed an increasing trend with temperature, with the highest activity observed at 41 °C, which was significantly higher than that at 35 °C. AchE activity peaked at 38 °C, where it was significantly higher than the control and other temperature treatments, but returned to control levels at 41 °C (Figure 6C). Total protein, triglyceride, trehalose, sorbitol and glycogen contents showed no significant changes among the different temperature treatments (Figure 6D). Overall, the larvae responded to high temperature mainly by enhancing antioxidant and detoxification enzyme activities, while energy metabolism and basic protein content remained stable (Figure 6).

### 3.7. Screening and Validation of Core Genes Involved in High-Temperature Stress

**Figure 7:**
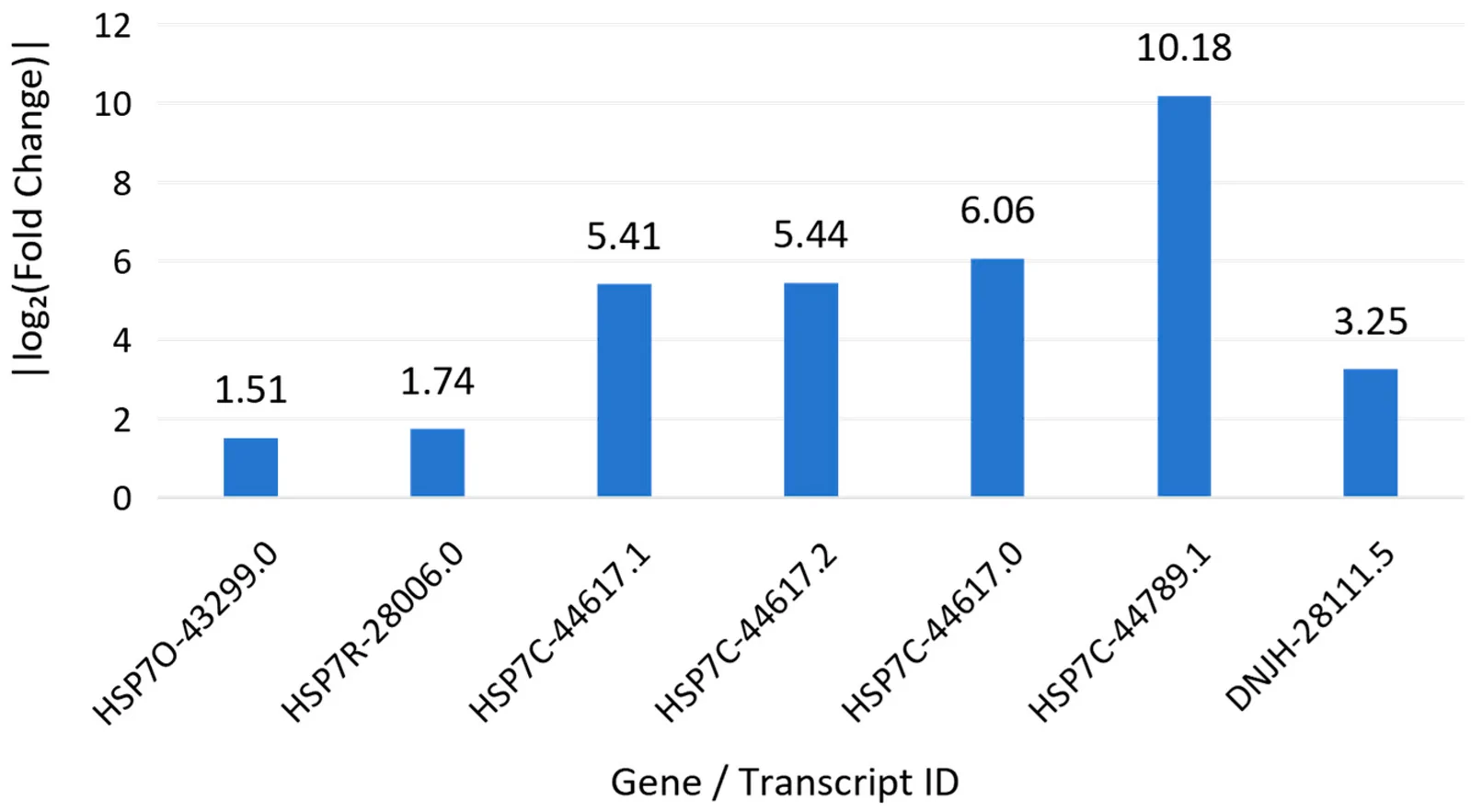
Expression changes in significantly down-regulated HSP family transcripts under 41 °C stress. All transcripts shown were significantly down-regulated (padj < 0.05). Numbers above bars indicate |log_2_FC| values.

Systematic analysis of the heat shock protein (HSP) family revealed divergent transcriptional responses under 41 °C stress. Only a subset of HSP transcripts displayed significant differential expression, and all of these regulated transcripts were down-regulated (Figure 7). Specifically, four transcripts annotated as *HSP7C*, one *HSP7R* transcript and one *HSP7O* transcript (partial members of the HSP70 family), together with the HSP40 homolog *DNJH*, were significantly down-regulated. By contrast, transcripts annotated as HSP80 exhibited no significant expression changes. This selective transcriptional repression targeting certain HSP70 members and HSP40 chaperones, instead of universal suppression across the entire HSP repertoire, suggested that 41 °C may exceed the effective induction threshold of canonical heat shock responses in *C. sacchariphagus* larvae.

**Figure 8:**
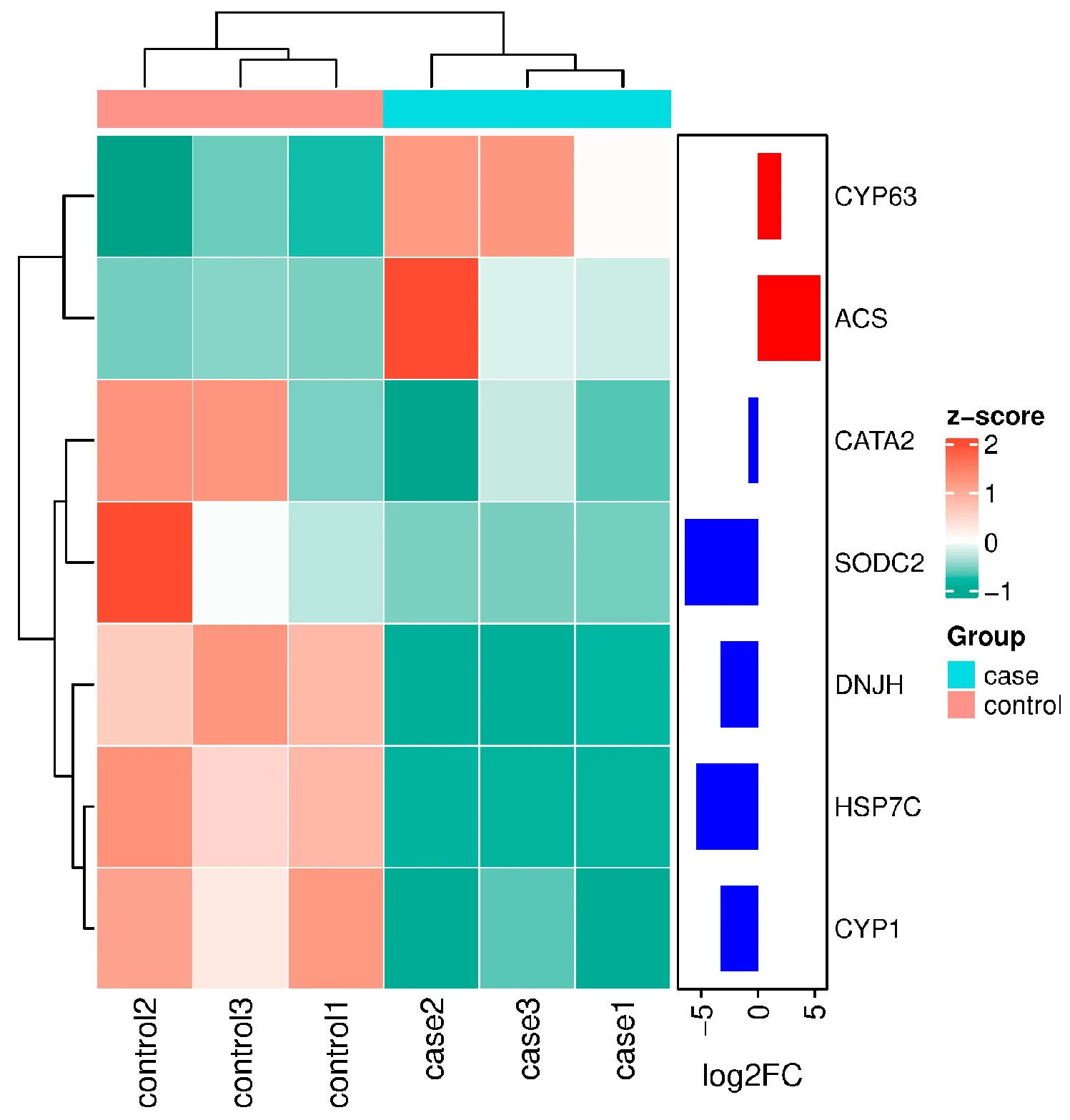
Heatmap of core genes involved in high-temperature stress response. Each row represents a core gene, each column a sample. Color scale from blue to red corresponds to low to high expression levels (Z-score normalized TPM values). Cases 1–3: 41 °C treatment; controls 1–3: 26 °C control. The bar on the right indicates log_2_FC values (red = up-regulated, blue = down-regulated).

Based on differential expression fold changes, functional enrichment and known biological relevance to stress responses, seven core genes were identified: heat shock proteins (*HSP7C*, *DNJH*), antioxidant (*SODC2*), energy metabolism (*ACS*), a cyclophilin gene with two transcripts (*CYP1* and *CYP63*, originating from the same gene locus Cluster-35096), and catalase (*CATA2*). Among these, *CATA2* was included as a representative of the catalase family because it was the only unigene confidently annotated as a catalase in our transcriptome, despite showing no significant differential expression. Under high-temperature stress, *ACS* (log_2_FC = +5.49) and *CYP63* (log_2_FC = +2.01) were significantly up-regulated, while *HSP7C* (log_2_FC = −5.44), *DNJH* (log_2_FC = −3.25), *SODC2* (log_2_FC = −6.46) and *CYP1* (log_2_FC = −3.32) were significantly down-regulated; *CATA2* (log_2_FC = −0.83) showed no significant change (Figure 8). Notably, CYP1 and CYP63 represent two transcript variants from the same gene locus, showing opposite expression patterns under heat stress (*CYP1* is down-regulated, *CYP63* is up-regulated).

**Figure 9:**
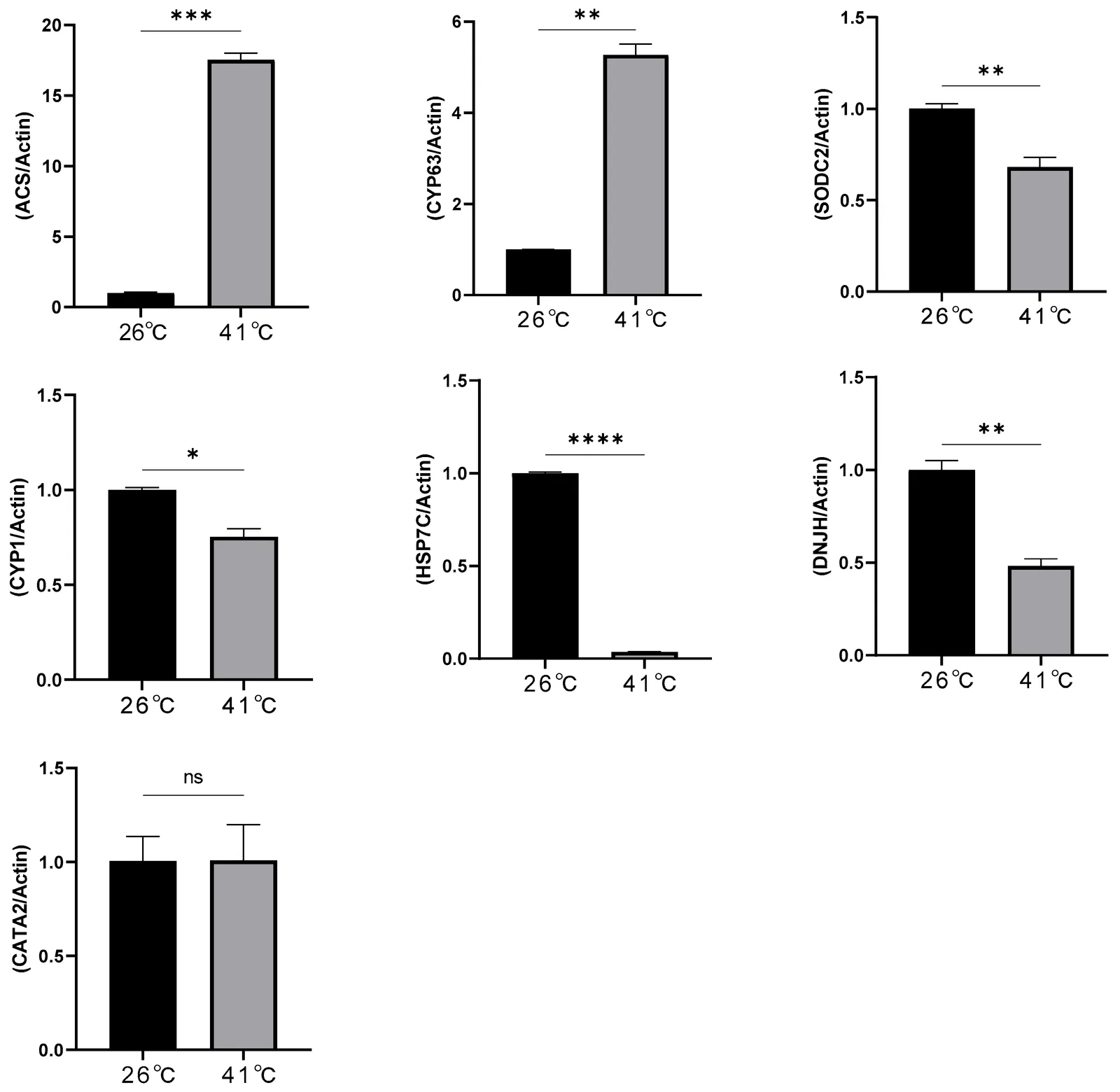
qRT-PCR validation of core gene expression under 41 °C heat stress vs. 26 °C control. The Y-axis represents relative target gene mRNA expression normalized to *Actin*, and the X-axis shows the 26 °C control and 41 °C treatment groups. ns = no significant difference (*p* > 0.05). Data are means ± SEM (*n* = 3). * *p* < 0.05, ** *p* < 0.01, *** *p* < 0.001, **** *p* < 0.0001.

To validate the transcriptome data, the seven core genes were examined by qRT-PCR. The results were highly consistent with the transcriptome data: *ACS* and *CYP63* were significantly up-regulated (*p* < 0.01); *HSP7C*, *DNJH*, *SODC2* and *CYP1* were significantly down-regulated (*p* < 0.05); and *CATA2* showed no significant change (*p* > 0.05) (Figure 9). The annotation information for the major genes discussed throughout this study is provided in Supplementary Table S1.

### 3.8. Association Analysis of Physio-Biochemical Indices with Transcriptome Data

To explore the potential molecular basis underlying these physio-biochemical responses, we compared each physiological indicator with the expression profiles of its corresponding genes. For most measured indices, physiological alterations were generally consistent with the transcriptional patterns of their encoding genes. However, several discrepancies were observed for CAT, SOD, CarE, and AchE. Specifically, CAT activity increased markedly at 41 °C, whereas the annotated catalase gene *CATA2* showed no significant transcriptional change (log_2_FC = −0.83). SOD activity peaked at 38 °C and then declined at 41 °C, accompanied by significant down-regulation of *SODC2* (log_2_FC = −6.46). For CarE and AchE, enzyme activities increased obviously under thermal stress, yet no differentially expressed genes were detected for their corresponding genes. POD activity was transiently activated at 35 °C, but no transcriptional data were available at this temperature. At 41 °C, among the unigenes annotated as peroxidases (based on the presence of the *An_peroxidase* domain, PF03098.18), only one gene (Cluster-44392.0) was significantly down-regulated (log_2_FC = −2.23), while the remaining peroxidases showed no significant changes. The integrated correlation results are summarized in Table 6. These discrepancies between enzyme activity and gene expression suggest that additional regulatory mechanisms may be involved in modulating these physiological responses under heat stress.

## 4. Discussion

In this study, transcriptome sequencing of fifth-instar *C. sacchariphagus* larvae exposed to 41 °C for 12 h revealed 8563 differentially expressed genes (DEGs), of which 3352 were up-regulated and 5211 down-regulated. This result indicates that high-temperature stress triggers extensive transcriptomic reprogramming involving numerous genes. Similar observations have been reported in other insects. In *Plutella xylostella* (Linnaeus, 1758) subjected to 40 °C, comparative transcriptome analysis identified 1253 DEGs [26]. In *Spodoptera frugiperda* (J.E. Smith, 1797), transcriptome analysis of adults after multigenerational heat selection also revealed hundreds of DEGs [22]. Collectively, these findings demonstrate that the insect response to high-temperature stress is a complex, polygenic process involving the coordinated regulation of multiple biological pathways.

The endoplasmic reticulum (ER) is an important organelle for protein synthesis and folding, and its homeostasis plays a critical role in the response to high-temperature stress. In our study, KEGG enrichment analysis pointed strongly to the “protein processing in endoplasmic reticulum” pathway, and ER homeostasis genes of the HRD family *HRD1B* (log_2_FC = −6.21) and *HRD3A* (log_2_FC = −1.50) were significantly down-regulated in the transcriptome, suggesting that ER function may be affected under heat stress. In the nematode *Caenorhabditis elegans* (Maupas, 1900), moderately elevated temperature activates the ER’s unfolded protein response (UPR), which through the IRE-1/XBP-1 pathway specifically represses ribosome biogenesis, alters lipid metabolism, and consequently boosts innate immunity [41]. In the small hive beetle *Aethina tumida* (Murray, 1867), lysosome-related pathways were significantly enriched under heat stress, indicating that autophagy and protein degradation play key roles in heat adaptation [42]. Functional annotation of the *C. sacchariphagus* transcriptome was performed using homology-based BLASTX searches against the NR database. While this approach provides valuable functional insights, it carries the inherent limitation of potential cross-kingdom annotation transfer, as the NR database contains sequences from diverse taxa. Therefore, species-mismatched GO terms and KEGG pathways (e.g., plant-specific terms) were excluded from biological interpretation.

Heat shock proteins (HSPs) are core molecular chaperone systems that enable cells to cope with high-temperature stress. In our study, multiple HSP70 family members (*HSP7C*, *HSP7R*, and *HSP7O*) and the HSP40 member *DNJH* were significantly down-regulated, while HSP80 (a member of the HSP90 family) and another HSP70 isoform (*HSP7M*) showed no significant changes. This pattern differs from the classical heat shock response, in which HSPs are usually up-regulated. It has become clear that HSP70 expression is temperature-dependent. In *Tetranychus truncatus* (Ehara, 1956), five HSP70 genes were significantly up-regulated under heat stress at 38–42 °C [21]. In *Sitodiplosis mosellana* (Géhin, 1857), expression of SmHSP70 peaked at 40 °C but was no longer significantly induced at 50 °C [43]. Similarly, in *B*. *tabaci*, hsp70 expression reached its maximum at 41 °C and then decreased rapidly at 43 °C and 45 °C [20], supporting the view that HSP70 expression has an optimal temperature range. In *Spodoptera litura* (Fabricius, 1775), Hsp70 interacts with the ecdysone receptor EcR and translocates from the cytoplasm to the nucleus under heat stress to participate in signaling regulation [44].The pronounced down-regulation of HSP70 family members and the HSP40 co-chaperone *DNJH* at 41 °C suggests that, in our experimental system, this temperature may exceed the effective induction range of the heat shock response in *C. sacchariphagus* larvae, potentially leading to a phase of heat damage. Moreover, the down-regulation of *DNJH*, a co-chaperone of HSP70, may further exacerbate the functional impairment of the protein folding system. Taken together, the systemic transcriptional suppression of HSP70 and HSP40 families observed in this study is consistent with a pattern of broad inhibition rather than activation at 41 °C. However, because transcriptome analysis was performed only at 26 °C and 41 °C, whether this temperature definitively exceeds the protective range of the heat shock response remains a hypothesis that warrants further investigation. Nevertheless, this pattern suggests that under our experimental conditions, 41 °C may have exceeded the effective induction range of the heat shock response and may have entered a phase of heat damage.

One of the most striking findings of this study is the “compensatory activation co-existing with basal functional inhibition” pattern in energy-metabolism-related genes. On one hand, the lipolysis key gene *ACS* (acetyl-CoA synthetase) was significantly up-regulated (log_2_FC = +5.49), indicating that the insect tried to cope with the increased energy demand under heat stress by enhancing lipid metabolism. On the other hand, genes involved in energy reserves such as glycogen and trehalose did not change significantly, and the overall pattern was one of “compensatory activation”. Similar results have been reported in *Loxostege sticticalis* (Linnaeus, 1761), where 30 °C accelerated adult emergence and ovarian development while shortening the oviposition period and adult lifespan; transcriptome analysis revealed significant up-regulation of energy-metabolism-related genes [45]. A metabolomics study of *Propylea japonica* (Thunberg, 1781) showed that lipid metabolites accounted for as much as 35.5% of all metabolites, and lipid remodeling and energy metabolic reprogramming are core strategies for heat adaptation [46]. In addition, Léger et al. studied three insect species—*Apis mellifera* (Linnaeus, 1758), *Drosophila melanogaster* (Meigen, 1830) and *Leptinotarsa decemlineata* (Say, 1824)—and found that under high temperature, the activities of enzymes related to FADH_2_ oxidation (CII and mG3PDH) were significantly enhanced, whereas NADH production was restricted, suggesting a conserved adaptation mechanism shifting energy metabolism towards FADH_2_ substrates [47]. Consistent with this, total protein and energy reserve substances (triglyceride, trehalose, sorbitol, glycogen) did not change significantly in our study, indicating that larvae maintained basic metabolic homeostasis under this acute heat stress regime.

Concurrently, the antioxidant system also plays an important role in the response to high-temperature stress. Catalase (CAT) activity increased sharply at 41 °C. However, the annotated catalase genes *CATA2* (log_2_FC = −0.83) showed no significant transcriptional changes under heat stress. Two additional catalase genes (Cluster-29223.0 and Cluster-16427.0) were also identified in the transcriptome, but they were not present in the differentially expressed gene list, suggesting that the increase in CAT activity may involve post-translational regulation or other mechanisms rather than transcriptional activation. This pattern of differential regulation among catalase isoforms has not been extensively documented in insect heat-stress studies. In *Tuta absoluta* (Meyrick, 1917), exposure to 44 °C for 3 h significantly reduced female adult survival, but the activities of three antioxidant enzymes (CAT, POD, SOD) did not change significantly, suggesting that its thermotolerance does not rely on the traditional antioxidant system [48]. In the two-spotted spider mite *Tetranychus urticae* (Koch, 1836), two catalase genes exhibited opposite expression patterns under heat stress—*TuCAT1* was up-regulated while *TuCAT2* was down-regulated, and RNAi silencing of *TuCAT1* decreased heat tolerance [24]. POD activity was transiently activated at 35 °C, but no transcriptional data were available at this temperature. At 41 °C, among the unigenes annotated as animal peroxidases (based on the An_peroxidase domain, PF03098.18), only one (Cluster-44392.0) was significantly differentially expressed, and it was down-regulated (log_2_FC = −2.23); the remaining peroxidases showed no significant changes. Thus, the elevated POD activity at 35 °C could not be explained by transcriptional up-regulation of peroxidase genes based on the current dataset. SOD activity peaked at 38 °C and then decreased slightly at 41 °C, while its encoding gene *SODC2* was significantly down-regulated (log_2_FC = −6.46) at 41 °C relative to the control, suggesting that the change in SOD activity may be regulated by post-translational or other mechanisms rather than at the transcriptional level. This is consistent with findings in *T*. *urticae*, where RNAi-mediated silencing of *TuSOD2* led to 87% mortality under high temperature, confirming the critical role of SOD in thermotolerance [49]. In *T. urticae*, heat stress also induced complex expression patterns of POD genes [50], which is similar to the response features of POD and SOD observed in our study. Collectively, these findings further reveal the regulatory diversity of the insect antioxidant system, showing that different insect species employ distinct strategies to modulate antioxidant enzyme activity under stress.

Detoxification enzymes also responded: CarE activity showed an increasing trend with temperature, and AchE activity was extremely significantly increased at 38 °C (about 2.2-fold of the control), reflecting the larval detoxification and neuroprotection mechanisms under high temperature. However, no significant differential expression of CarE or AchE encoding genes was detected in the 41 °C transcriptome. This discrepancy between enzyme activity and transcript abundance may be attributable to post-translational modifications, enhanced protein stability, or other mechanisms such as post-transcriptional regulation and changes in protein turnover. It is also possible that transcriptional activation of CarE and AchE occurs at lower temperatures (e.g., 35–38 °C) and becomes suppressed under prolonged 41 °C stress, resembling the behavior of the HSP70 family; however, in the absence of transcriptomic data at intermediate temperatures, this remains speculative. In *Cyrtorhinus lividipennis* (Reuter, 1885), high-temperature stress similarly induced marked changes in energy-metabolism- and stress-response-related genes [51]. Meanwhile, total protein and energy reserve substances did not change significantly, and the expression of trehalose metabolism-related genes *TPS* (log_2_FC = −0.39) and *TRE* (log_2_FC = −0.08) also remained unchanged, consistent with the stable trehalose content. This result differs from the significant increase in trehalose content under high-temperature stress reported in *C. lividipennis* [51], possibly reflecting species- or developmental-stage-specific strategies for coping with high-temperature stress. GST activity showed no significant differences among temperatures, and the expression of its encoding genes also showed no significant changes, indicating transcription-function consistency.

Collectively, our study describes the transcriptional and physiological responses of heat-stressed *C. sacchariphagus* larvae, including selective suppression of HSP70/40 chaperones and increased activities of antioxidant and detoxification enzymes without corresponding transcriptional changes. These findings provide a basis for understanding how this pest responds to extreme heat at both transcriptional and physiological levels. While these findings are informative, several limitations of this study should be acknowledged. Our analyses were confined to fifth-instar larvae under an acute heat exposure regime, which does not capture the gradual temperature fluctuations that insects naturally encounter. In addition, the physio-biochemical assays were performed across a temperature gradient (26, 35, 38, and 41 °C), whereas RNA-seq was only conducted on the control (26 °C) and the most extreme heat-stressed (41 °C) groups. Consequently, the observed peaks of POD activity at 35 °C and AchE activity at 38 °C could not be directly correlated with transcriptomic changes at those specific temperatures. Moreover, although catalase (CAT) activity increased at 41 °C, none of the annotated catalase genes showed transcriptional changes under heat stress, suggesting that the increase may involve post-translational regulation or other mechanisms, but this requires further investigation. Furthermore, RNAi-mediated functional validation of the seven core genes would provide direct causal evidence for their roles in heat tolerance. Addressing these questions in future work will further advance our understanding of the thermal biology of *C. sacchariphagus*.

## 5. Conclusions

Transcriptomic and physio-biochemical analyses of heat-stressed fifth-instar *C. sacchariphagus* larvae revealed 8563 differentially expressed genes involved in protein homeostasis, stress signaling, and energy metabolism. HSP70/40 family members were significantly down-regulated, while HSP80 remained stable. Catalase activity increased at 41 °C, but none of the annotated catalase genes showed transcriptional changes. CarE and AchE activities also increased under heat stress, yet no corresponding transcriptional changes were detected for their encoding genes. Seven genes (*ACS*, *HSP7C*, *DNJH*, *SODC2*, *CYP1*, *CYP63*, and *CATA2*) are suggested as candidates for future functional studies. These findings describe the transcriptional and physiological responses of *C. sacchariphagus* larvae to extreme heat, although the underlying mechanisms require further investigation.

## Figures and Tables

**Table 1:** Primer sequences used in qRT-PCR.

Gene	Primer Sequence (5′ → 3′)	
*DNJH*	F	ATCCTCCTCGTAAGCGTTGC
	R	TCCTAATCGCATTCCGCCTG
*HSP7C*	F	GCCTAAACGTGCTTCGCATC
	R	GTGTATCTCCGGCAGTAGCC
*SODC2*	F	GGAAACCATGCAAGCCCTTC
	R	TGCGTTCTCAATGGGGATGT
*ACS*	F	ACGAAAGGCGATGTTCCACT
	R	GACCGATAAGGTGACGCACT
*CYP1*	F	TCTCTGCTGTTTTGGGCACTA
	R	AATGATACCAGGCCACTGCTC
*CYP63*	F	CTTATGAGGCACGGAGGAGC
	R	CAGTTGTTAGCGCATCGGAG
*CATA2*	F	TAGTTGGGGCAACCCTCTTG
	R	GTCATCGTCTGGGAGCGAAT
*Actin*	F	CAATCCTAAAGCCAACAGA
	R	GCGTAGCCCTCGTAGAT

**Table 2:** Summary of transcriptome sequencing data quality (provided by the sequencing facility).

Sample	Raw Reads	Raw Data (G)	Clean Reads	Clean Data (G)	Effective (%)	Error (%)	Q20 (%)	Q30 (%)	GC (%)
case1	64428452	9.7	63516406	9.5	98.58	0	99.34	96.24	45.00
case2	65944004	9.9	64994802	9.7	98.56	0	99.37	96.51	45.03
case3	40382750	6.1	39804488	5.9	98.57	0	99.50	97.35	45.30
control1	57812590	8.7	56883676	8.5	98.39	0	99.48	97.13	44.70
control2	60294836	9.0	59418122	8.9	98.55	0	99.46	96.97	45.13
control3	61591550	9.2	60658370	9.1	98.48	0	99.44	96.94	45.40

**Table 3:** Statistics of transcriptome assembly.

Index	All	Average Length	Median Length	Total Assembled Bases	N50
Transcript	132,531	1222.42	656	162,008,603	2069
Unigene	90,394	1054.94	546	95,359,879	1812

**Table 4:** Statistics of unigene functional annotation.

Database	Number of Annotated Unigenes	Percentage (%)
Total unigenes	89,352	100%
Total transdecoder peptides	52,380	58.6%
SwissProt-BLASTX	5440	6.1%
SwissProt-BLASTP	4833	5.4%
PFAM	15,545	17.4%
NR	21,790	24.4%
eggNOG	3251	3.6%
KEGG	1548	1.7%
GO	10,943	12.3%

**Table 5:** Statistics of differentially expressed genes.

Comparison	Significantly Up-Regulated Genes	Significantly Down-Regulated Genes	Total
Control vs. case	3352	5211	8563

**Table 6:** Summary of association between physio-biochemical indices and gene expression.

Index	Physio-Biochemical Result	Corresponding Gene Expression at 41 °C
CAT	Significantly increased at 41 °C	*CATA2* (log_2_FC = −0.83) unchanged
SOD	Peaked at 38 °C then declined	*SODC2* (log_2_FC = −6.46) significantly down-regulated
POD	Peaked at 35 °C	No up-regulated peroxidases detected; one peroxidase (Cluster-44392.0 *, log_2_FC = −2.23) was down-regulated
CarE	Showed increasing trend, peaked at 41 °C	No differentially expressed genes detected
AchE	Extremely increased at 38 °C	No differentially expressed genes detected
GST	No significant change	Most members unchanged
Trehalose	No significant change	*TPS* (log_2_FC = −0.39), *TRE* (log_2_FC = −0.08) unchanged
Total protein	No significant change	Overall expression stable
TG	No significant change	*ACS* (log_2_FC = +5.49) up-regulated but TG unchanged
Sorbitol	No significant change	No differentially expressed genes detected
Glycogen	No significant change	No differentially expressed genes detected

* Cluster-44392.0 was annotated as a peroxidase based on the presence of the *An_peroxidase* domain (PF03098.18).

## Data Availability

The raw sequence data reported in this paper have been deposited in the Genome Sequence Archive (Genomics, Proteomics & Bioinformatics 2025) in National Genomics Data Center (Nucleic Acids Res 2025), China National Center for Bioinformation/Beijing Institute of Genomics, Chinese Academy of Sciences (GSA: CRA047502, accessed on 1 August 2026) that are publicly accessible at https://ngdc.cncb.ac.cn/gsa.

## Supplementary Materials

The following supporting information can be downloaded at: https://www.mdpi.com/article/10.3390/insects17090907/s1, Table S1: Annotation information of core genes discussed in this study.
